# The Quality Assessment of Virtual Unenhanced and Blending Images Derived from Dual-Energy CT for Detecting Colorectal Cancer

**DOI:** 10.2174/0115734056412910251125054025

**Published:** 2026-01-30

**Authors:** Feixiang Chen, Weize Xu, Jianfeng Zhu, Meirong Wang, Jinghao Chen, Jing Xiao, Jushun Yang, Bosheng He

**Affiliations:** 1 Department of Radiology, Affiliated Hospital 2 of Nantong University, Nantong, Jiangsu 226001, China; 2 Department of Radiology, Affiliated Hospital of Nantong University, Nantong, Jiangsu 226001, China; 3 Department of Epidemiology and Medical Statistics, School of Public Health, Nantong University, Nantong 226019, China; 4 Translational Medicine Research Center, Affiliated Hospital 2 of Nantong University, Nantong 226001, China

**Keywords:** Colorectal cancer, Computed tomography, Virtual unenhanced, Blending image, Linear blending and non-linear blending images

## Abstract

**Introduction:**

This study aimed to evaluate the image quality of virtual unenhanced and blending images from dual-energy CT for detecting colorectal cancer (CRC).

**Materials and Methods:**

A total of 72 patients with pathologically diagnosed CRC underwent abdominal dual-energy CT, following which virtual unenhanced, linear blending, and non-linear blending images were generated by post-processing reconstruction. Both subjective and objective evaluations were conducted on these images, with signal-to-noise (SNR) and contrast-to-noise ratio (CNR) calculations conducted for organs, such as the liver, pancreas, and spleen.

**Results:**

Virtual unenhanced images of CRC, extraserosal fat of the tumor, liver, pancreas, spleen, kidney, and subcutaneous fat showed a lower signal intensity than both linear and non-linear blending images (*P* < 0.05), while the CNR of virtual unenhanced images was higher than linear and non-linear blending images (*P* < 0.05). Except for CRC lesions, the SNR of other organs in virtual unenhanced images was higher than in linear and non-linear blending images (*P* < 0.05). There were no significant differences in subjective image scores and the number of conventional lesions between virtual unenhanced image, linear, and non-linear blending (*P* ≥ 0.05). The Kappa coefficients for evaluating extraserosal invasion were 0.722, 0.584, and 0.584 for virtual unenhanced, linear blending, and non-linear blending images, respectively, with corresponding accuracies of 86.1%, 79.2%, and 79.2%.

**Conclusion:**

Virtual unenhanced images of patients with CRC can provide high-quality images for diagnostic evaluation, potentially replacing linear blending and non-linear blending images in plain scans.

## 1 BACKGROUND/INTRODUCTION

Colorectal cancer (CRC) is a prevalent malignant tumor of the digestive system and has been a significant global economic burden [1-3]. It ranks third in incidence and second in mortality among malignant tumors worldwide [4, 5]. In 2020, the global burden of CRC is projected to increase by 60%, resulting in over 2.2 million new cases and 1.1 million deaths [6]. Surgical resection effectively cures the majority of patients with early-stage primary CRC, with around 90% achieving successful outcomes [7, 8]. Unfortunately, a significant number of CRC cases are diagnosed at advanced stages, leading to distant organ metastasis and precluding the possibility of radical surgery [9]. Staging assessment plays a vital role in determining the severity and extent of spread of CRC, guiding physicians in selecting appropriate treatment plans [10]. Therefore, early diagnosis and accurate clinical staging of CRC are crucial.

Computed tomography (CT) is a noninvasive technique for preoperative staging evaluation of cancer patients [11-13]. The National Comprehensive Cancer Network (NCCN) and the Clinical Practice Guidelines for Colon Cancer by the Union for International Cancer Control (UICC) have incorporated CT into the routine examination for colon cancer screening, preoperative imaging assessment, and the evaluation of adjuvant chemotherapy in high-risk populations [14]. Extramural invasion (EMI) is an important prognostic factor of colon cancer. The EMI sensitivity detected by multidetector CT is 81% and 87% in patients with CRC, the specificity is 50% and 75%, and the positive rate of EMI is 91.5%, indicating that multidetector CT can reliably detect T3/T4 tumors [15]. CT can detect T3-T4 tumors with strong sensitivity; its sensitivity and specificity are 80% and 76%, respectively, and the accuracy is 79% [16]. The overall diagnostic sensitivity of CT to calculate the T stage of these pT4 tumors is 56.9%; however, the accuracy of preoperative staging of T3 and T4 tumors is 89.8% [17]. CT demonstrated the accuracy to be increased by 23% in T staging of CRC, and specificity increased to 67.1% in N staging [18].

Dual-energy CT takes advantage of the different attenuation properties of X-rays at varying energy levels as they pass through the same material, enabling effective material differentiation [19-21]. This technology allows for dual-energy scanning in conventional plain scans and generates linear blending images and non-linear blending images [22]. In abdominal plain scans, the linear blending image from a dual-energy scan, with a mixing coefficient of 0.5, can effectively replace the conventional plain scan 120 kVp image in daily work. Research reports that the image quality of non-linear blending images surpasses that of linear blending images [23], and the overall image quality of virtual unenhanced images is comparable to that of conventional plain scans [24]. Plain scan images serve as the foundation for abdominal examination in patients with CRC, aiding in the evaluation of staging and other conditions. While previous studies have explored the potential of dual-energy CT in CRC detection, this study aimed to provide a more nuanced evaluation by comparing virtual unenhanced images not only with conventional blending images but also by assessing their diagnostic accuracy in detecting extraserosal invasion. Our findings may offer valuable insights for optimizing preoperative staging and treatment planning in patients with CRC.

## 2 METHODS

### 2.1 Study Design

This study was a prospective experimental study aimed at evaluating the image quality of virtual unenhanced images, linear blending images, and non-linear blending images derived from dual-energy CT for the detection of CRC. The objective was to explore the potential clinical value of virtual unenhanced images as a substitute for traditional blending images in diagnostic evaluations. Patients with pathologically diagnosed CRC underwent abdominal dual-energy CT, after which virtual unenhanced, linear-blending, and non-linear-blending images were generated *via* post-processing reconstruction. Both subjective and objective evaluations were conducted on these images, with signal-to-noise (SNR) and contrast-to-noise ratio (CNR) calculations conducted for organs, such as the liver, pancreas, and spleen.

The study followed the Sex and Gender Equity in Research guidelines (SAGER) to ensure appropriate inclusion and reporting of sex-related data.

### 2.2 Patients

Patients with CRC underwent surgical resection, and staging was confirmed by pathology in Affiliated Hospital 2 of Nantong University from January 2020 to May 2021. This study was approved by the ethics committee (approval number: 2020YKS024) and conducted strictly in accordance with the World Medical Association guidelines of the Declaration of Helsinki.

Inclusion criteria were as follows: (a) preoperative patients who had undergone a full abdominal dual-energy CT plain scan before surgery, with the diagnosis confirmed by surgery and pathology within two weeks; (b) no other malignant tumors; (c) no neoadjuvant therapy, such as radiotherapy or chemotherapy, performed before the operation; (d) no serious heart, lung, or kidney diseases affecting image evaluation; (e) no obvious motion artifacts affecting image observation.

Exclusion criteria were as follows: (a) no full abdominal dual-energy CT enhanced scanning or enhanced CT examination performed, or the interval was more than 2 weeks; (b) imaging examination affected by severe underlying disease; (c) patients having tumor recurrence or received neoadjuvant therapy, such as radiotherapy or chemotherapy, before the operation; (d) postoperative pathology having confirmed adenoma, neuroendocrine tumor, stromal tumor, or inflammation; (e) patients with iodine contrast agent allergy or renal insufficiency (creatinine > 120 μmol/L).

### 2.3 CT Protocols and Image Acquisition

All CT scans were performed utilizing the SIEMENS SOMATOM Force open-source CT machine. The patient followed a fluid diet the day before the examination and slowly ingested 2000 mL of 2.5% isotonic mannitol orally within 2 hours of the examination. The patient was positioned supine with a scanning range from the top of the diaphragm to the pubic symphysis. The voltage of tube A was set at 90 kV with a tube current-time product of 144 mAs. Tube B had a voltage of 150 kV with a tube current-time product of 90 mAs. The pitch was 1.0, the speed was 0.5 seconds, and the collimation width was 2 × 96 × 0.6 mm. Reconstruction utilized the Br36 convolutional accounting method with a layer thickness of 1 mm and an interval of 1 mm. Enhanced scanning used the Bolus tracking method to monitor the aorta at the level of the thoracic 11 vertebral body. The arterial phase was triggered five seconds after the aorta reached a monitoring threshold of 100 HU, followed by the venous phase after 40 seconds. The contrast medium with iodoprolamine (370 mgI/mL) was injected through the elbow vein at a dose of 1.5 mL/kg and an injection rate of 3.5 mL/s, followed by rinsing with 20-30 mL of physiological saline. In addition, the non-contrast CT scan linear blending image had a mixing coefficient of 0.5 (50% each for 90 KV and Sn150 KV). Reconstruction layer thickness was 1.0 mm with a spacing of 1 mm. In the optimum contrast mode of the Siemens Syngovia post-processing workstation, λ=50 HU and ω=200 HU were selected for the non-linear fusion image, and the thickness of the reconstructed layer was 1.0 mm, and the layer spacing was 1 mm.

#### 2.3.1 Virtual Unenhanced Image

The dual-energy arterial phase image was selected, and the Live VNC mode of the dual-energy interface was activated. The iodine blending ratio was adjusted to 0 to obtain the virtual unenhanced image, with a reconstruction layer thickness of 1.0 mm and a layer spacing of 1 mm. The linear blending, non-linear blending, and virtual unenhanced images were then reconstructed into cross-sectional images with a layer thickness of 3 mm and a layer spacing of 3 mm.

### 2.4 Quantitative Image Analysis

The quality score of the image was jointly evaluated by doctors with 8 and 12 years of diagnostic experience, who conducted blind evaluation for the image and clinical information on virtual unenhanced, linear blending, and non-linear blending images, both subjectively and objectively.

#### 2.4.1 Subjective Evaluation

Subjective evaluation was scored using the 5-point scoring standard [25]. A score of 5 indicated excellent image quality, precise depiction of anatomical structures, clear identification of lesion boundaries, and facilitation of accurate and clear diagnosis. A score of 4 indicated good image quality, clear anatomical structures and lesion boundaries, and a small number of artifacts, not affecting the diagnosis. A score of 3 indicated most anatomical structures to be relatively clear, details not being clearly displayed, and the noise being high, but generally acceptable. A score of 2 indicated unclear display of anatomical structures, poor image quality, and significant noise. A score of 1 indicated extremely poor image quality, large artifacts, blurry organizational structure, and an inability to evaluate the images. The image quality score of 3 or above could meet clinical diagnostic needs. Disagreements in the scoring were resolved through mutual consultation.

#### 2.4.2 Objective Evaluation

Regions of interest (ROI) were drawn on images for CRC lesions, liver, pancreas, spleen, kidney, rectal tumors, extraserosal fat of rectal tumors, and subcutaneous fat at the tumor level. Care was taken to avoid blood vessels, calcifications, and necrotic areas. Liver ROI area was about 1 cm^2^, while the ROI area of pancreas, spleen, kidney, and subcutaneous fat was approximately 0.5 cm^2^. The area of interest of CRC lesions was limited to two-thirds of the overall thickness of the bowel wall lesion. Consistency in the size, shape, and position of the ROI in each group of images was maintained [26]. CT values and standard deviation (SD) of the ROI were recorded, with the subcutaneous fat SD value at the tumor level used as the noise reference. SNR and CNR were calculated according to the following formulas: SNR = CT value of lesion/SD, and CNR = (CT value of lesion - CT value of fat)/noise. The above values were measured three times, and the averaged results were recorded (the measurements of all objective indicators of the virtual unenhanced images were measured using the Siemens Syngovia post-processing workstation). The number of lesions observed on imaging examinations was recorded, including conventional low-density lesions in the liver, spleen, and kidneys, gallbladder stones, and other tumor lesions. The difference in lesions among the three sets of images was compared. Pathological results regarding tumor location (low, medium, and high rectal cancer, colon cancer) and extraserosal invasion were recorded, and the consistency evaluation of extraserosal invasion in the three sets of images was conducted. This multi-faceted approach ensured a comprehensive evaluation of image quality and diagnostic performance.

### 2.5 Statistical Analysis

All statistical analyses were conducted using SPSS 26.0 software. Continuous variables with a normal distribution have been represented as mean ± standard deviation (SD), while those without a normal distribution have been represented as median ± interquartile spacing. The objective quality indicators, such as lesions and parenchymal organs, in virtual unenhanced, linear blending, and non-linear blending images were compared using one-way ANOVA for normally distributed data, with pairwise comparison using the S-N-K method. Non-normally distributed data were analyzed using nonparametric rank tests and the Kruskal-Wallis H test, followed by the Bonferroni method for inter-group comparisons. Subjective image scores and the number of conventional lesions in diagnostic results were compared by nonparametric rank and the Kruskal-Wallis H test. Extraserosal invasion of tumors in the three image sets and pathological extraserosal invasion were compared using the Kappa consistency test, with consistency scores categorized as follows: 0-0.20 for extremely low consistency, 0.21-0.40 for average consistency, 0.41-0.60 for moderate consistency, 0.61-0.80 for high consistency, and 0.81-1.00 for almost complete consistency. *P* < 0.05 was considered to indicate a statistically significant difference.

## 3 RESULTS

### 3.1 Patient Demographics

A total of 98 consecutive patients with suspected colorectal mass were enrolled; of these, 26 cases were excluded because they did not meet the inclusion criteria. These included one case with contrast agent allergy, two cases with renal insufficiency, four cases without abdominal enhanced CT examination, three cases with an interval between surgical examination and image examination of more than two weeks, four cases with poor image quality due to motion artifacts or underlying diseases, and two cases with tumor recurrence. Three patients received preoperative neoadjuvant therapy, four patients were diagnosed as having adenoma, 1 patient was diagnosed with a neuroendocrine tumor, one patient was diagnosed with a stromal tumor, and one patient was diagnosed with inflammation after the operation. Finally, 72 patients were enrolled, including 44 males and 28 females, with a mean age of 65.4 ± 9.27 years (range: 43-88 years) (Table **S1**). This final cohort included patients with colon cancer (n = 27), high rectal cancer (n = 15), median rectal cancer (n = 21), and low rectal cancer (n = 9). There were a total of 40 cases located above the peritoneal reflection, 13 cases located across the peritoneal reflection, and 19 cases under the peritoneal reflection.

Notably, our cohort comprised patients at different stages of CRC, providing a comprehensive evaluation of virtual unenhanced images across diverse clinical scenarios.

### 3.2 Subjective Evaluation of Image Quality

As shown in Table **1**, the subjective scores of virtual unenhanced, linear blending, and non-linear blending image groups exceeded 3 points, meeting the requirements for clinical diagnosis. More exactly, the subjective scores of virtual unenhanced, linear blending, and non-linear blending image groups were 4.72 ± 0.61, 4.76 ± 0.59, and 4.76 ± 0.59 points, respectively. There was no statistically significant difference in subjective scores among the three groups (*P* > 0.05) (Table **1**, Figs. (**1**-**4**).

### 3.3 Objective Evaluation of Image Quality

The objective evaluation of image quality was also conducted (Table **2**). The results showed that the virtual unenhanced images of CRC, extraserosal fat of the tumor, liver, pancreas, spleen, kidney, and subcutaneous fat exhibited lower noise than the linear and non-linear blending image groups (*P* < 0.05). The CNR of CRC, extraserosal fat of the tumor, liver, pancreas, spleen, and kidney in the virtual unenhanced image was higher than in linear and non-linear blending images (*P* < 0.05). Except for CRC lesions, the SNR of extraserosal fat of the tumor, liver, pancreas, spleen, and kidney in virtual unenhanced images was higher than that in linear and non-linear blending images (*P* < 0.05).

### 3.4 Comparison of Image Diagnostic Indicators

**Fig. (1):**
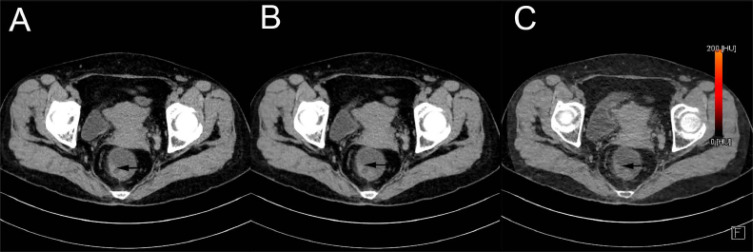
A 61-year-old female patient with median rectal cancer. (**A**) Non-linear blending image (subjective score: 4 points). (**B**) Linear blending image (subjective score: 4 points). (**C**) Virtual unenhanced image (subjective score: 3 points). The black hollow tail arrow indicates the location of the tumor, and the white solid tail arrow indicates the tumor invasion of the serosa.

**Fig. (2):**
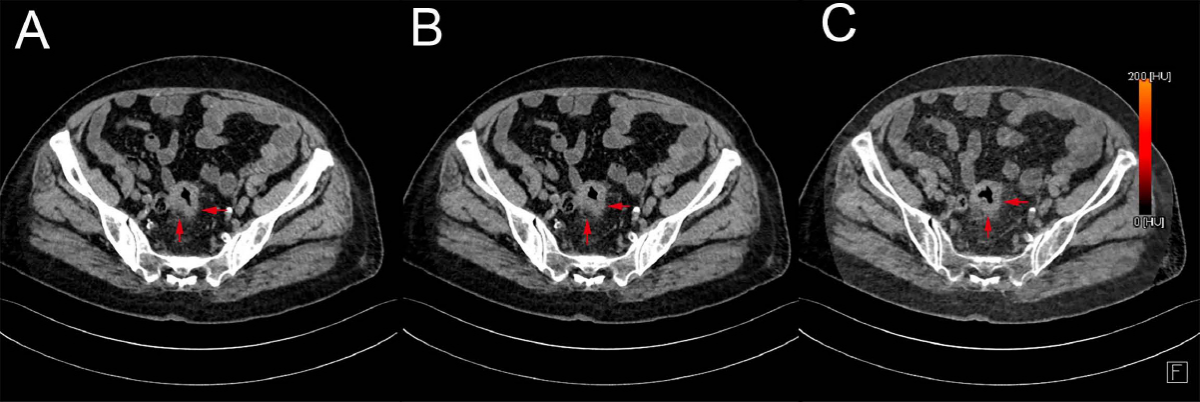
A 73-year-old female patient with high rectal cancer. (**A**) Non-linear blending image (subjective score: 5 points). (**B**) Linear blending image (subjective score: 5 points). (**C**) Virtual unenhanced image (subjective score: 5 points). The white hollow tail arrow indicates the location of the tumor, and the white solid tail arrow indicates the tumor invasion of the serosa.

**Fig. (3):**
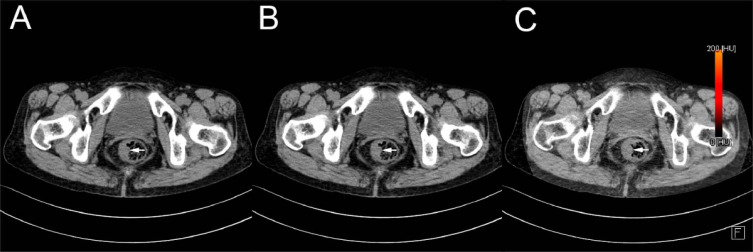
A 71-year-old female patient with low rectal cancer. (**A**) Non-linear blending image (subjective score: 5 points). (**B**) Linear blending image (subjective score: 5 points). (**C**) Virtual unenhanced image (subjective score: 5 points). The white hollow tail arrow indicates the location of the tumor.

**Fig. (4):**
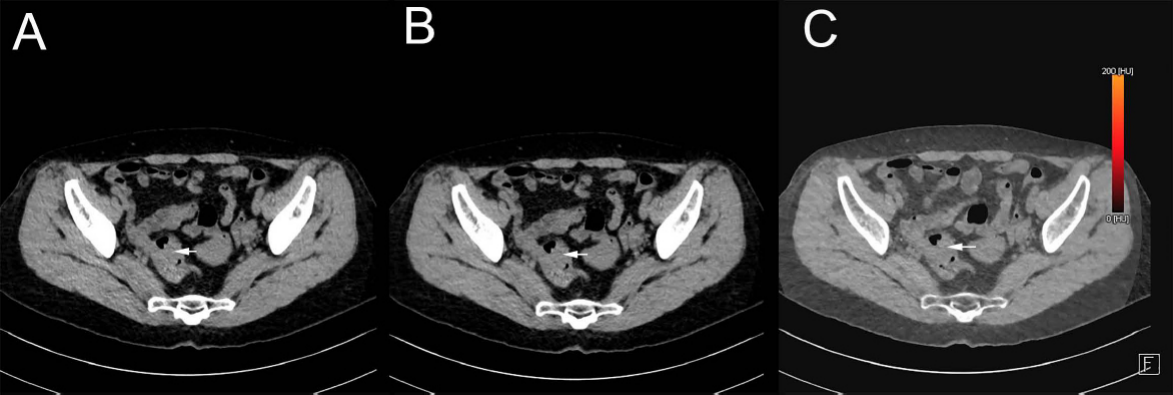
A 64-year-old female patient with sigmoid colon cancer. (**A**) Non-linear blending image (subjective score: 5 points). (**B**) Linear blending image (subjective score: 5 points). (**C**) Virtual unenhanced image (subjective score: 5 points). The white hollow tail arrow indicates the location of the tumor.

The common lesions displayed in virtual unenhanced images, linear blending, and non-linear blending images were evaluated Table **3**. There was no significant statistical difference in the visualization of liver low-density lesions, kidney low-density lesions, spleen low-density lesions, gallbladder stones, kidney stones, and lymph nodes around tumors among virtual unenhanced, linear blending, and non-linear blending images (*P* > 0.05). Notably, two cases of kidney stones smaller than 2 mm were visible in both linear and non-linear blending images, but not in virtual unenhanced images; one negative gallbladder stone was not found in linear and non-linear blending images, but was visible in virtual unenhanced images. Additionally, the study explored the use of virtual unenhanced, linear blending, and non-linear blending images for assessing extraserosal invasion and surgical pathology consistency. The Kappa coefficients of virtual unenhanced, linear blending, and non-linear blending images for evaluating extraserosal invasion were 0.722, 0.584, and 0.584, respectively; the corresponding accuracies were 86.1%, 79.2%, and 79.2%, respectively (Table **4**, Figs. **1**-**4**). The corresponding coronal and sagittal images had the same effect (Figs. **S1**-**S4**).

## 4 DISCUSSION

CRC staging and grading information play a crucial role in guiding treatment decisions and predicting patient outcomes. Tailoring treatment to specific cancer stages can lower the risk of recurrence and metastasis post-surgery [27-29]. In view of a certain lag in pathological diagnosis, imaging evaluation has emerged as a crucial foundation for selecting preoperative personalized treatment plans, assessing postoperative efficacy, and predicting survival outcomes in CRC [30-32]. Dual-energy CT colonography has demonstrated a 95% accuracy rate in diagnosing colon cancer when compared to histopathological findings, while also minimizing acquisition time, examination costs, and patient discomfort [33]. Furthermore, although MRI exhibits high accuracy in the preoperative staging of rectal cancer, it does present a certain false positive rate when excluding lymph node metastasis, primarily due to the influence of the crossing of small blood vessels in the abdomen. Additionally, MRI is limited in its application for patients with metal implants [34]. Conventional CT examinations typically necessitate multi-phase scanning, which results in a relatively high radiation dose. In contrast, dual-energy CT employs a material separation post-processing algorithm to distinguish iodine from fat and soft tissue, producing virtual non-enhanced images. This technique effectively reduces the radiation dose for patients without compromising lesion visualization [35].

Previous studies have indicated that although the image quality of virtual unenhanced scans may be marginally inferior to that of traditional plain scans, it remains sufficient for diagnostic requirements [24, 36]. Consistent with these findings, this study found that the subjective scores of groups' virtual unenhanced, linear blending, and non-linear blending images were 4.72 ± 0.61, 4.76 ± 0.59, and 4.76 ± 0.59 points, respectively. This study found no significant difference in the number of low-density lesions in the liver, spleen, and kidney, as well as gallbladder stones and bilateral kidney stones, among common abdominal lesions. However, a few cases showed that virtual unenhanced scans could not clearly display punctate urinary tract stones seen in linear and non-linear blending images. This may be due to the incomplete removal of iodine in organs and tissues, which may have affected the detection of small rocks and calcification [37]. A few cases showed that virtual unenhanced images could clearly display gallbladder stones not seen in linear or non-linear blending images. Traditional CT images often display isodensity or low density, making it challenging to differentiate them from bile [38]. Studies have also found that virtual unenhanced images are more sensitive than conventional non-contrast for detecting a few low-density lesions, especially small cysts and necrotic liquefaction areas. This enhanced sensitivity is achieved through improved scanning intensity and the strategic application of contrast agents [39]. Overall, the virtual unenhanced images fulfill diagnostic requirements and are suitable for clinical diagnosis. Additionally, virtual unenhanced images minimize radiation exposure during conventional plain scanning for patients. A research work reported that dual-energy CT virtual unenhanced imaging in patients with rectal cancer reduced the effective dose in the arterial and venous phases by approximately 34% compared to conventional plain scans [40, 41]. Therefore, virtual unenhanced imaging not only meets diagnostic image quality standards, but also effectively reduces radiation exposure.

This study distinguished itself from prior research through several key innovations. First, it employed a multi-dimensional evaluation framework combining subjective image quality assessments by experienced radiologists with objective quantitative metrics and pathology-correlated diagnostic accuracy for extraserosal invasion. Second, the cohort design was uniquely representative, encompassing patients across a broad age range and diverse tumor locations, ensuring generalizability. Third, while previous studies have focused on image quality, our analysis quantified diagnostic consistency between virtual unenhanced CT and surgical pathology, demonstrating superior accuracy in staging compared to blending images. Finally, the radiation dose reduction achieved by virtual unenhanced protocols has addressed a critical unmet need in CRC surveillance. By integrating these advancements, this study has provided robust evidence for clinical adoption of virtual unenhanced CT, offering a safer, more accurate alternative for preoperative staging and treatment planning.

This study demonstrated that virtual unenhanced images of CRC as extraserosal fat of the tumor, liver, pancreas, spleen, kidney, and subcutaneous fat exhibited lower noise than linear and non-linear blending in image groups. Additionally, the CNR of CRC as extraserosal fat of the tumor, liver, pancreas, spleen, and kidney in virtual unenhanced images was higher than that in linear and non-linear blending images. Except for CRC lesions, the SNR of extraserosal fat of the tumor, liver, pancreas, spleen, and kidney in virtual unenhanced images was higher than that in linear and non-linear blending images. These results have been found to be in line with the previous studies, which have reported lower noise in tumors and peritoneal fat in virtual unenhanced images compared to conventional plain scans, while observing higher CNR in virtual unenhanced CT scans [40, 42]. However, there was no significant difference in the tumor SNR between the virtual unenhanced images and the blending images in this study, possibly due to the heterogeneity of the tumor and the optimization of the blending images. Additionally, there was no significant difference between the non-linear blending image and the linear blending image, contradicting previous research findings [26]. Both the linear blending and non-linear blending images were derived from a plain scan image in this study, potentially due to inadequate blending width and the proportion of linear blending. The high contrast characteristics of non-linear blending may not have been fully utilized. Therefore, further research is needed to explore the impact of blending factors and parameters (λ and ω values) on the visualization of rectal cancer lesions.

The diagnosis of tumor extraserosal infiltration *via* CT primarily relies on extraserosal fat effusion, which is crucial for T2 and T3 staging. If the periintestinal fat space appears blurred and the outer edge of the intestinal wall is irregular, this is indicative of invasion. However, peritumoral inflammatory infiltration may present similar signs, potentially leading to an overestimation of extraserosal infiltration [43]. This study determined the consistency of virtual unenhanced, linear blending, and non-linear blending images in evaluating tumor extraserosal invasion and correlating with pathological results to be 0.722, 0.584, and 0.584, respectively, with an accuracy of 86.1%, 79.2%, and 79.2%, respectively. The study revealed the consistency and accuracy of virtual unenhanced diagnosis of extraserosal invasion to be significantly higher than blending images. The study also revealed the coincidence rate of dual-energy CT virtual non-contrast scans in the T staging of rectal cancer to be 83%. Specifically, the coincidence rates for T1-T2, T3, and T4 stages were 91.1%, 83.3%, and 92.2%, respectively. This improvement may be primarily attributed to dual-energy CT's ability to enhance the contrast between the lesion and the surrounding soft tissue, thereby facilitating the assessment of serosal surface invasion and improving staging accuracy [44]. Furthermore, the material separation techniques used in virtual unenhanced images help differentiate exudate from inflammatory infiltration, potentially avoiding overestimation [45, 46].

However, it was important to acknowledge the limitations of virtual unenhanced (VUE) images in detecting very small lesions, such as minute kidney stones. Due to the inherent limitations in spatial resolution and CNR of VUE images compared to conventional enhanced CT scans, there may be instances where very small lesions, particularly those with low attenuation differences from surrounding tissues, are not clearly visualized. This was evident in a few cases within our study, where punctate urinary tract stones smaller than 2 mm were not consistently visible on VUE images, although they were detectable on linear and non-linear blending images. This limitation underscores the need for cautious interpretation of VUE images in clinical scenarios where the detection of small lesions is critical. Future advancements in CT technology, including improvements in spatial resolution and algorithms for material decomposition, may help mitigate this limitation and enhance the diagnostic utility of VUE images across a broader spectrum of lesion sizes.

Recent advancements in surgical technology have underscored the transformative potential of the internet of things (IoT) in CRC care. IoT-enabled devices, such as smart sensors embedded in surgical instruments, wearable patient monitors, and connected endoscopic systems, facilitate real-time data acquisition and transmission during CRC diagnosis, treatment, and follow-up [47]. IoT-integrated laparoscopic tools can transmit intraoperative parameters to enhance surgical precision in minimally invasive CRC resections. Similarly, IoT-based postoperative monitoring systems enable continuous tracking of recovery metrics, allowing for timely intervention in cases of anastomotic leaks or infections, which are common complications in CRC surgery. Furthermore, IoT platforms support telemedicine applications, enabling remote postoperative follow-up and patient education, which is particularly valuable for rural or underserved populations. While our study has focused on imaging-based diagnosis, the integration of IoT with dual-energy CT or other diagnostic modalities could enable automated image analysis, real-time alerts for suspicious lesions, and seamless data sharing across multidisciplinary CRC care teams. However, challenges, such as data security, interoperability, and clinical validation, remain hurdles for widespread IoT adoption in CRC management. Future research should explore synergies between IoT and imaging technologies to optimize CRC outcomes across the care continuum.

## 5 STUDY LIMITATIONS

There were numerous limitations in this study. Firstly, the sample size was relatively small, and a larger sample size is needed to avoid bias. Secondly, there may have been deviations in this study due to the inability to completely maintain a uniform size during the ROI drawing process. Thirdly, this study focused on image quality, with no additional T or N staging studies conducted. Lastly, the virtual unenhanced images generated during the arterial phase were selected for evaluation without confirming any differences between the virtual unenhanced images of the arterial and venous phases. Given these limitations, including the relatively small sample size and potential variability in pathological staging, our results should be interpreted with caution. While the promising performance of virtual unenhanced images in detecting extraserosal invasion (pT4) is encouraging, the preliminary nature of our findings necessitates validation through larger, multicenter studies with more diverse patient populations. Future research should aim to incorporate a greater number of cases across all pT stages, particularly focusing on extraserosal invasion, to confirm the robustness and generalizability of our observations.

## CONCLUSION

In conclusion, virtual unenhanced images are superior to linear and non-linear blending images in the process of colorectal cancer examination, particularly in terms of lower noise, higher CNR, and improved diagnostic consistency for extraserosal invasion, which underscores their potential as a replacement for conventional blending images in preoperative colorectal cancer staging. They have the potential to replace blending images, reduce the radiation dose, and improve the consistency and accuracy of diagnosis in extraserosal invasion. This study can help enhance the diagnostic value of colorectal cancer.

## Figures and Tables

**Table 1:** Subjective evaluation of image quality.

**Group**	**Linear Blending Image**	**Non-linear Blending Image**	**Virtual Unenhanced Image**
Image score	4.76±0.59	4.76±0.59	4.72±0.61
Z-value (*P*-value)	-	0.498 (0.78)	-

**Table 2:** Objective evaluation of image quality.

**Item**	**Virtual Unenhanced Image**	**Linear Blending Image**	**Non-linear Blending Image**	**Z-value/F-value**	***P*-value**	**Linear Blending *vs.* non-linear Blending**	**Linear Blending *vs.* Virtual unenhanced**	**Non-linear Blending *vs.* Virtual Unenhanced**
Liver noise	8.30±1.08	10.75±3.13	11.80±3.25	107.235	<0.001	0.158	<0.001	<0.001
Liver SNR	7.20±1.96	5.663±1.64	5.164±2.02	62.792	<0.001	0.414	<0.001	<0.001
Liver CNR	8.14±2.42	5.33±2.32	5.32±2.62	68.769	<0.001	1.000	<0.001	<0.001
Pancreas noise *	9.70±1.69	12.56±2.43	12.94±2.40	46.665	<0.001	0.308	<0.001	<0.001
Pancreas SNR	4.36±1.67	3.85±1.32	3.65±1.21	17.115	<0.001	1.000	0.002	0.001
Pancreas CNR*	5.63±1.82	3.82±1.41	3.97±1.46	29.225	<0.001	0.570	<0.001	<0.001
Spleen noise	7.60±1.40	9.85±2.90	10.25±3.40	87.220	<0.001	0.302	<0.001	<0.001
Spleen SNR	6.62±1.81	5.63±2.06	5.29±1.61	33.085	<0.001	0.504	<0.001	<0.001
Spleen CNR	6.60±1.89	4.77±1.89	4.88±1.26	51.487	<0.001	1.000	<0.001	<0.001
Renal noise	8.25±1.35	10.45±2.98	11.25±3.60	76.971	<0.001	0.394	<0.001	<0.001
Renal SNR	3.97±1.42	3.40±1.07	3.27±1.18	14.911	<0.001	1.000	0.011	0.001
Renal CNR	3.98±1.20	2.70±1.33	2.90±1.67	38.066	<0.001	0.597	<0.001	<0.001
Tumor noise	9.00±2.78	11.05±4.53	12.45±4.60	41.976	<0.001	0.403	<0.001	<0.001
Tumor SNR	4.00±1.37	3.78±1.63	3.3±1.30	11.685	0.003	0.329	0.208	0.002
Tumor CNR	4.41±1.54	3.21±1.59	3.35±1.63	41.456	<0.001	1.000	<0.001	<0.001
Tumor extraserosal fat noise	11.80±4.05	16.70±6.05	16.35±5.35	55.810	<0.001	1.000	<0.001	<0.001
Tumor extraserosal fat SNR	3.72±3.75	3.87±3.29	3.72±3.77	11.685	0.003	0.329	0.208	0.002
Tumor extraserosal fat CNR	3.59±4.94	3.42±4.55	3.13±5.23	41.456	<0.001	1.000	<0.001	<0.001
Subcutaneous fat noise	6.65±1.30	9.60±3.48	9.50±3.23	102.607	<0.001	1.000	<0.001	<0.001

**Note:** SNR: Signal-to-noise ratio; CNR: Contrast-to-noise ratio. *represents that the data in this group followed a normal distribution.

**Table 3:** Evaluation of common lesion display in images.

**Item**	**Virtual Unenhanced Image**	**Linear Blending Image**	**Non-linear Blending Image**	**Z-value/F-value**	***P*-value**	**Linear Blending *vs.* Non-linear Blending**	**Linear Blending *vs.* Virtual Unenhanced**	**Non-linear Blending *vs.* Virtual Unenhanced**
Liver low-density lesion	0.96±1.96	0.96±1.96	0.96±1.96	0.000	1.000	-	-	-
Renal low-density lesion	0.97±1.80	0.97±1.80	0.97±1.80	0.000	1.000	-	-	-
Spleen low-density lesion	0.08±0.44	0.06±0.37	0.06±0.37	0.294	0.863	-	-	-
Gallstone	0.19±0.46	0.18±0.45	0.18±0.45	0.065	0.968	-	-	-
Kidney stone	0.13±0.47	0.14±0.51	0.14±0.51	0.001	1.000	-	-	-
Periintestinal lymph nodes	1.72±1.84	1.72±1.84	1.72±1.84	0.000	1.000	-	-	-

**Table 4:** Comparison of consistency between images of extraserosal invasion and surgical pathology.

**Group**	**Image Assessment of Extraserosal Invasion**	**Surgical Pathology Extraserosal Invasion**		**Kappa Coefficient**	**Accuracy**
		**yes**	**no**		
Virtual unenhanced image	yes	31	6	0.722	86.1%
	no	4	31		
Linear blending image	yes	29	9	0.584	79.2%
	no	6	28		
Non-linear blending image	yes	29	9	0.584	79.2%
	no	6	28		

## Data Availability

The data and supportive information are available within the article.

## LIST OF ABBREVIATIONS

CRC: Colorectal cancer
CT: Computed tomography
NCCN: National Comprehensive Cancer Network
UICC: Union for International Cancer Control
EMI: Extramural invasion
CNR: Contrast-to-noise ratio
IoT: Internet of things

## STANDARDS OF REPORTING

STROBE guidelines were followed.
