# Supplementary material for: The Quality Assessment of Virtual Unenhanced and Blending Images Derived from Dual-Energy CT for Detecting Colorectal Cancer
Source: Curr Med Imaging. 2026 Jan 30;22:e15734056412910. doi: 10.2174/0115734056412910251125054025 (PMC13312390; doi:10.2174/0115734056412910251125054025)
Supplement: Supplementary file 1 [file CMIM-22-E15734056412910_SD1.pdf]

1875-6603/26 Send Orders for Reprints to reprints@benthamscience.net

Current Medical Imaging

Content list available at: https://benthamscience.com/journals/cmir

Supplementary Material

The Quality Assessment of Virtual Unenhanced and Blending Images Derived from Dual-Energy CT for Detecting Colorectal Cancer

Feixiang Chen[1], Weize Xu[1], Jianfeng Zhu[1], Meirong Wang[1], Jinghao Chen[2], Jing Xiao[3], Jushun Yang[1,*] and Bosheng He[1,4,*]

[1]*Department of Radiology, Affiliated Hospital 2 of Nantong University, Nantong, Jiangsu226001, China*
[2]*Department of Radiology, Affiliated Hospital of Nantong University, Nantong, Jiangsu226001, China*
[3]*Department of Epidemiology and Medical Statistics, School of Public Health, Nantong University, Nantong 226019, China*
[4]*Translational Medicine Research Center, Affiliated Hospital 2 of Nantong University, Nantong 226001, China*

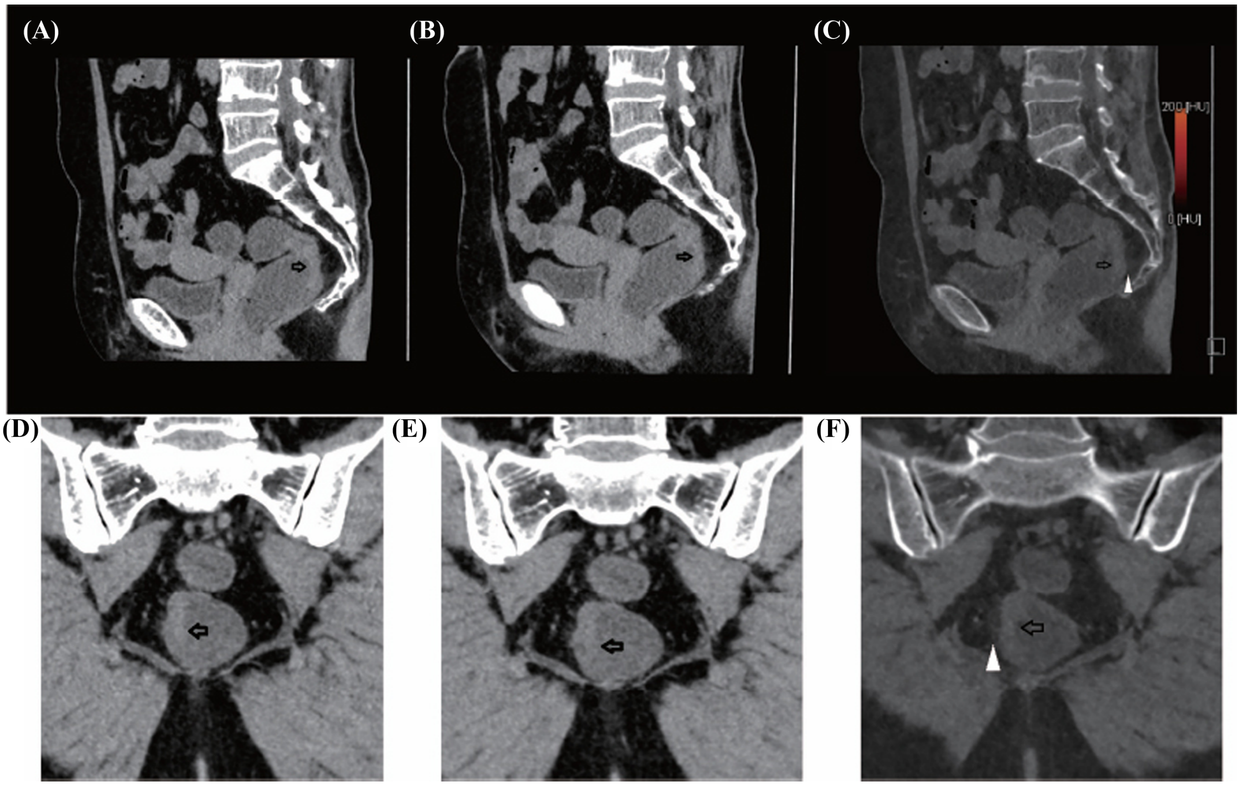

Fig. (S1). The sagittal (**A**-**C**) and coronal images (**D**-**F**) of a 61-year-old female patient with median rectal cancer. (**A**) Non-linear blending sagittal image (subjective score: 4 points). (**B**) Linear blending sagittal image (subjective score: 4 points). (**C**) Virtual unenhanced sagittal image (subjective score: 3 points). (**D**) Non-linear blending coronal image (subjective score: 4 points). (**E**) Linear blending coronal image (subjective score: 4 points). (**F**) Virtual unenhanced coronal image (subjective score: 3 points). The black hollow tail arrow indicates the location of the tumor, and the white solid tail arrow indicates the tumor invasion of the serosa.

DOI: 10.2174/0115734056412910251125054025, 2026, *22*, e15734056412910

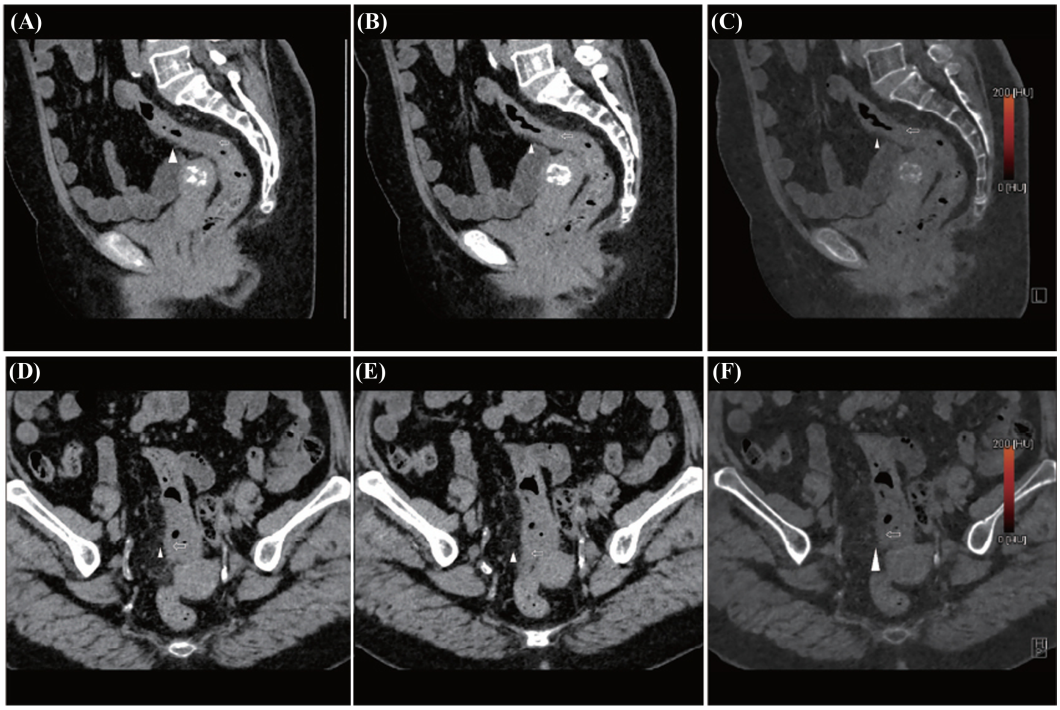

Fig. (S2). The sagittal (**A**-**C**) and coronal images (**D**-**F**) of a 73-year-old female patient with high rectal cancer. (**A**) Non-linear blending sagittal image (subjective score: 5 points). (**B**) Linear blending sagittal image (subjective score: 5 points). (**C**) Virtual unenhanced sagittal image (subjective score: 5 points). (**D**) Non-linear blending coronal image (subjective score: 5 points). (**E**) Linear blending coronal image (subjective score: 5 points). (F) Virtual unenhanced coronal image (subjective score: 5 points). The white hollow tail arrow indicates the location of the tumor, and the white solid tail arrow indicates the tumor invasion of the serosa.

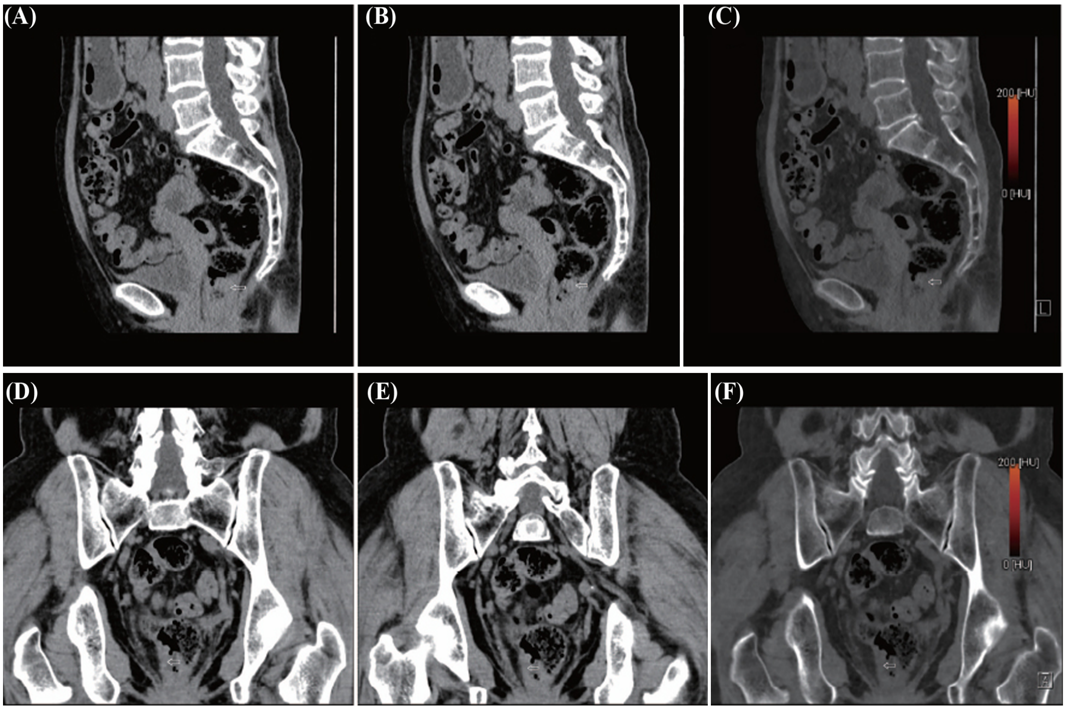

Fig. (S3). The sagittal (**A**-**C**) and coronal images (**D**-**F**) of a 71-year-old female patient with low rectal cancer. (**A**) Non-linear blending sagittal image (subjective score: 5 points). (**B**) Linear blending sagittal image (subjective score: 5 points). (**C**) Virtual unenhanced sagittal image (subjective score: 5 points). (**D**) Non-linear blending coronal image (subjective score: 5 points). (**E**) Linear blending coronal image (subjective score: 5 points). (**F**) Virtual unenhanced coronal image (subjective score: 5 points). The white hollow tail arrow indicates the location of the tumor.

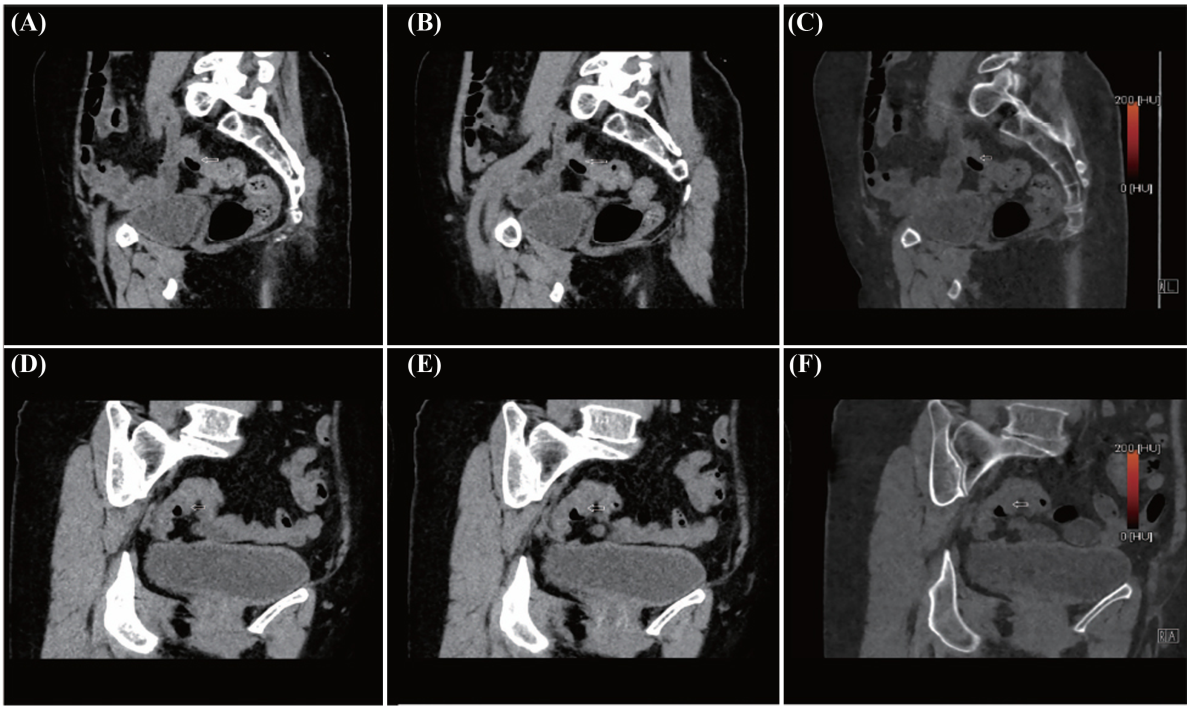

Fig. (S4). The sagittal (**A**-**C**) and coronal images (**D**-**F**) of a 64-year-old female patient with sigmoid colon cancer. (**A**) Non-linear blending sagittal image (subjective score: 5 points). (**B**) Linear blending sagittal image (subjective score: 5 points). (**C**) Virtual unenhanced sagittal image (subjective score: 5 points). (**D**) Non-linear blending coronal image (subjective score: 5 points). (**E**) Linear blending coronal image (subjective score: 5 points). (**F**) Virtual unenhanced coronal image (subjective score: 5 points). The white hollow tail arrow indicates the location of the tumor.

Table S1. Location, pathologic type and stage of the tumor.

Classification Type	Types	Number of Cases	Ratio
Tumor Location	Ascending Colon	6	0.0833
	Hepatic Flexure Of Colon	1	0.0139
	Colon Transversum	2	0.0278
	Splenic Flexure Of Colon	2	0.0278
	Colon Descendens	5	0.0694
	Sigmoid Colon	11	0.1528
	Rectal	45	0.625
Pathological Type	Adenomatous Carcinoma	64	0.8889
	Mucinous Adenocarcinoma	4	0.0556
	Signet-Ring Cell Carcinoma	1	0.0139
	Undifferentiated Carcinoma	1	0.0139
	Neuroendocrine Differentiated Adenocarcinoma	1	0.0139
	Squamous Differentiated Adenocarcinoma	1	0.0139

(Table S1) contd.....

Classification Type	Types	Number of Cases	Ratio
Pathological Stage	T1	9	0.125
	T2	28	0.3889
	T3	32	0.4444
	T4a	1	0.0139
	T4b	2	0.0278

© 2026 The Author(s). Published by Bentham Science Publisher.

This is an open access article distributed under the terms of the Creative Commons Attribution 4.0 International Public License (CC-BY 4.0), a copy of which is available at: https://creativecommons.org/licenses/by/4.0/legalcode. This license permits unrestricted use, distribution, and reproduction in any medium, provided the original author and source are credited.

HOW TO CITE:
Chen F, Xu W, Zhu J, Wang M, Chen J, Xiao J, Yang J, He B. The Quality Assessment of Virtual Unenhanced and Blending Images Derived from Dual-Energy CT for Detecting Colorectal Cancer. Curr Med Imaging, 2026; 22: e15734056412910.
http://dx.doi.org/10.2174/0115734056412910251125054025
